# A Novel Radar Sensor for the Non-Contact Detection of Speech Signals

**DOI:** 10.3390/s100504622

**Published:** 2010-05-04

**Authors:** Mingke Jiao, Guohua Lu, Xijing Jing, Sheng Li, Yanfeng Li, Jianqi Wang

**Affiliations:** Department of Biomedical Engineering, Fourth Military Medical University, Xian, 710032, China; E-Mails: jmkok@126.com (M.K.J.); lugh1976@fmmu.edu.cn (G.H.L.); fmmujxj@fmmu.edu.cn (X.J.J.); sheng@mail.xjtu.edu.cn (S.L.); yanfengli_0_0@yahoo.com.cn (Y.F.L.)

**Keywords:** non-contact detection, microwave radar, speech signal, penetrability

## Abstract

Different speech detection sensors have been developed over the years but they are limited by the loss of high frequency speech energy, and have restricted non-contact detection due to the lack of penetrability. This paper proposes a novel millimeter microwave radar sensor to detect speech signals. The utilization of a high operating frequency and a superheterodyne receiver contributes to the high sensitivity of the radar sensor for small sound vibrations. In addition, the penetrability of microwaves allows the novel sensor to detect speech signals through nonmetal barriers. Results show that the novel sensor can detect high frequency speech energies and that the speech quality is comparable to traditional microphone speech. Moreover, the novel sensor can detect speech signals through a nonmetal material of a certain thickness between the sensor and the subject. Thus, the novel speech sensor expands traditional speech detection techniques and provides an exciting alternative for broader application prospects.

## 1. Introduction

Obtaining accurate, reliable speech signals is necessary for humans to communicate and exchange information in various situations, especially in noisy environments. Numerous research groups have developed different techniques for detecting speech signals that can be obtained from the vibrational information of particles in an air medium or surface of a body caused by sound. Conventional speech and acoustic transducers, such as condenser microphones, detect speech signals by perceiving the motion of air particles when sound is spread via an air medium [1]. Another technique that has been thoroughly explored is speech detection via perceptions of sound pressure [2,3]. Li Zong Wen’s group reported using the Doppler radar with grating structures to detect speech signals [4]. They studied the operating principle based on the wave propagation theory and the interaction between the electromagnetic wave (EMW) and the acoustic wave (AW) on large numbers of particles in the air and on the interface of two media. Speech detection sensors that detect the vibrations of the speech organ and skin have been used clinical diagnoses [5,6], in measuring speech articulator motions [7–9], and speech recognition and encoding [10,11]. However, these previously developed sensors have potential limitations. Traditional speech and acoustic transducers have no penetrating power whereas speech signals produced from the vibrations of speech organs lose most of their high frequency components. Some sensors based vibration have to be in contact with the body of subjects throughout the procedure [12,13], which makes them feel nervous and uncomfortable. Speech quality and the requirements for detection are uncertain for low signal-to-noise and DC offsets using radars with grating structures because of receiver drawbacks.

A novel speech sensor that uses a millimeter microwave (MMW) radar with high operating frequency and a superheterodyne receiver has been developed. In many MMW radar systems, the high operating frequency and the superheterodyne receiver have been widely used for the detection of small displacements of vibration [14,15]. The high operating frequency can generate a larger modulated phase, which lends high sensitivity to small vibration displacements of high frequency speech [16]. The superheterodyne receiver can reduce DC offsets and 1/f noise [17], so that signal-to-noise ratio and the detection sensitivity for small high frequency speech signals are improved. Furthermore, since the microwave can penetrate some non-metal media, such as wood and glass, the radar can remotely sense vibrational information even when there are barriers between it and the subject [18,19].

This paper evaluates the use of the novel radar sensor as a potential tool for detecting speech signals. The novel sensor was used for speech detection in various conditions and radar speech quality was assessed via the spectrogram analyses of speech signals and Mean Opinion Score (MOS) tests [20,21]. Detailed comparisons between the novel sensor and traditional condenser microphones were performed. The ability of the radar sensor to penetrate non-metal materials was assessed as well.

## 2. Description of the MMW Radar Speech Sensor

**Figure 1.**
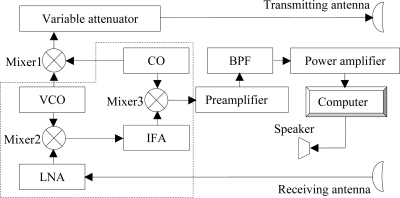
Block diagram of the radar speech sensor.

Figure 1 shows a block diagram of the novel radar speech sensor. The system is composed of oscillators, transmitters, a superheterodyne receiver, and baseband circuits. The volt control oscillator (VCO made of GaAs Gunn) operates at 34.5 GHz. It has a low noise, and a high frequency stability of 10 ppm. Here the frequency stability is the amount of frequency deviation from the assigned value over a specified period of time. The crystal oscillator (CO) generates a very stable sine signal of 1 GHz. The merging of VCO and CO signals produces a microwave signal of 35.5 GHz, with the beams being radiated by the transmitting antenna. The transmitting and receiving antennas are both parabolic, with a maximum antenna gain of 38.5 dB at 35.5 GHz and an estimated beam width of 9°. The antenna can reduce interferences from other directions and produce higher directivity gains against other antennas. A variable attenuator (0–35 dB) controls the power level of the transmitted electromagnetic wave signal. The superheterodyne receiver receives echo wave signals, including speech information through a low noise amplifier (LNA) [22]. It uses the principle of frequency mixing or heterodyning to convert the received signal to a lower intermediate frequency, which can be more conveniently processed than the original carrier frequency, thereby reducing DC offsets and 1/f noise [17]. The superheterodyne receiver, represented by the dashed box, includes two down-converters (Mixer2 and Mixer3), oscillators (VCO and CO), and amplifiers (LNA and intermediate frequency amplifier (IFA)). Speech signals exported from the superheterodyne receiver are processed by the baseband circuits, which consist of a traditional preamplifier, a band-pass filter (BPF, frequency from 100 Hz to 5,000 Hz) and power amplifiers. The final signals are sampled via a 16-channel A/D converter (USB7333; Zhongtai, Beijing; CHINA) to be transferred to a computer for further processing. Speech signals recorded by the computer can be played back through a speaker.

## 3. Signal Recording and Processing

**Figure 2.**
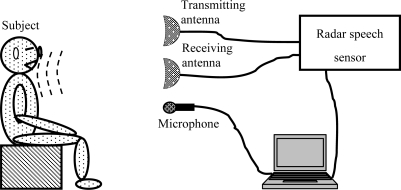
Diagram of experimental design for detecting speech signals.

The radar speech detection sensor and a traditional condenser microphone were positioned 4 m away from the subject (Figure 2), so that they can simultaneously collect speech signals from the subject. A distance of 4 m was chosen to enable the collection of high quality speech signals in a relatively quiet environment, although the novel sensor could detect speech signals that are 70 m away. The performances of the novel radar sensor and the microphone were evaluated by listening to computer recordings.

The speech detection capability of the proposed radar sensor through barriers was also evaluated. For standard speech material, phrases (“one two three four”) were recorded with a microphone in a quiet environment and saved as a WAV file. First, a loudspeaker was placed 4 m from the antenna of the novel radar sensor without barriers between, and the standard speech material was played over the loudspeaker to be detected by the radar sensor. The same procedure was performed with a 6-cm thick wooden door between the loudspeaker and the antenna. The third setup involved a 7-cm thick sandwich brick wall, composed of two 1-cm thick wood board with bricks between them, between the loudspeaker and the antenna.

Speech signals were sampled at a frequency of 10,000 Hz via a 16-channel A/D converter and recorded by a computer and saved as a text file for further processing using the MATLAB software package (MATLAB version 6.5; The Math Works, Inc; Natic, Massachusetts; USA).

Ten healthy volunteer speakers (10 males; 26.2 ± 5.0 years) participated in the experiments. All the experiments were conducted according to the terms of the Declaration of Helsinki (BMJ 1991; 302:1194), and all participants signed the appropriate consent forms.

### 3.1. Denoise of the speech signal recording

Speech signals recorded by either a traditional condenser microphone or a novel radar speech sensor, even in a relatively quiet environment, are usually contaminated by some background or electrocircuit noise. Thus, recorded noise was reduced using the spectral subtraction algorithm [23], which has been widely used in noise canceling and has been shown to be effective in improving the quality of speech. This method enhances speech signals by subtracting short-term average noise spectrum from the noisy speech spectrum. The noise spectrum is estimated during silence or no speech activity intervals from the input signal. If a speech signal *s* (*t*) is degraded by the uncorrelated additive noise signal *n* (*t*):

(1) y(t)=s(t)+n(t)

The short-term power spectrum of noisy speech can be approximated as:

(2) $${\left|Y(\omega )\right|}^{2}\approx {\left|S(\omega )\right|}^{2}+{\left|N(\omega )\right|}^{2}$$

where |*Y*(*ω*)|^2^, |*S*(*ω*)|^2^ and |*N*(*ω*)|^2^ represent the noisy speech short-term spectrum, the clean speech spectrum, and the noise power spectrum estimates, respectively. The estimates are obtained by replacing noise power |*N*(*ω*)|^2^ with its average value |*N*(*ω*)|*^γ^* taken during no speech activity intervals. For minimizing residual and musical noise, the generalized spectral subtraction scheme proposed by Berouti *et al.* [23] was employed:

(3) $${\left|\hat{S}(\omega )\right|}^{\gamma }=\{\begin{array}{ll} {\left|Y(\omega )\right|}^{\gamma }-\alpha {\left|\bar{N}(\omega )\right|}^{\gamma }, & \text{if}\frac{{\left|\bar{N}(\omega )\right|}^{\gamma }}{{\left|Y(\omega )\right|}^{\gamma }}<\frac{1}{\alpha +\beta }\\ \beta {\left|\bar{N}(\omega )\right|}^{\gamma }, & \text{otherwise}, \end{array}$$

where *α* (*α* > 1) is the over-subtraction factor, *β* (0 ≤ *β* ≤ 1) is the spectral floor, and *γ* is the exponent factor of transition grade. Values were set as *γ* = 2, and *β* = 0.002, while *α* can be adjusted according to different speech conditions to obtain better speech quality. Enhanced speech signals were obtained using the power spectrum of enhanced speech and the phase of the input signals [24].

Finally, speech reproduced by the novel radar sensor was evaluated using a spectrogram [25], which is a visual representation of speech energy distribution across frequencies and over time. It can identify the strength and frequencies of formants, and can pick out individual harmonics. The spectrogram results were examined manually to identify energy distributions, which were then used to compare the speech signals from the traditional microphone and the proposed novel radar sensor.

### 3.2. Coherence analysis of speech signals

Differentiation of varying sounds lies in their characteristic chord, which is composed of a fundamental frequency (F0) and a harmonic [26]. Therefore, speech is most closely related to frequency, and different speech signals have different frequency components. Pitch and frequency are directly related, such that a high pitch has a high frequency and a low pitch has a low frequency. Coherence analysis was used to estimate the strength of correlation of the frequency domains of the speech signals from the traditional microphone and the radar sensor that were recorded simultaneously from the same speaker [27].

The squared coherence spectrum function *C*xy(*ω*) for the traditional condenser microphone speech signal *x* and the novel radar sensor speech signal *y* is defined as [28]:

(4) $$C\text{xy}(\omega )=\frac{{\left|\mathrm{Pxy}(\omega )\right|}^{2}}{\mathrm{Pxx}(\omega )\mathrm{Pyy}(\omega )}$$

where *Pxx*, *Pyy*, and *Pxy* represent the power spectral densities of *x* and *y*, and the cross power spectral density of *x* and *y*, respectively. *C*xy(*ω*) is a function of frequency with values between 0 and 1 that indicates how well the novel radar sensor speech signal *y* corresponds to the traditional microphone speech signal *x* at each frequency. The higher the amplitude of the coherence spectra, the better the coherence between the two types of speech signals.

To determine the corresponding relationship between the radar speech sensor and the traditional microphone speech, coherence was calculated and plotted for frequencies from 0 to 5 kHz. Signal coherence analysis and confidence levels were accomplished with the software package MATLAB using its signal analysis and statistics toolbox (MATLAB version 6.5; The Math Works, Inc; Natic, Massachusetts; USA).

### 3.3. MOS test of speech signal

The MOS test is the simplest numerical method of speech quality evaluation [29–31]. Instruction sheets with a five-point scale (1: bad; 2: poor; 3: common; 4: good; 5: excellent) were prepared for 48 listeners to measure speech quality based on MOS criteria. The listeners were asked to listen to 20 sentences recorded simultaneously by the novel radar speech sensor and the traditional microphone and to evaluate them using the scale provided. They were divided into eight equal groups, with each group evaluating the same material. The average scores of the radar sensor speech and the traditional microphone speech from each group were calculated. All listeners (48 males; 30.2 ± 3.6 years) are healthy and have no reported history of hearing problems. The tests were performed in a soundproof room with a high quality headphone and a comfortable loudness (60 dB sound pressure level (SPL)).

## 4. Experimental Results

**Figure 3.**
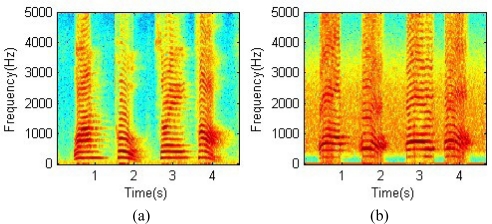

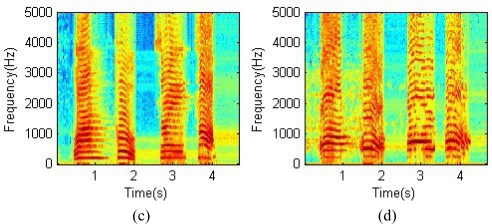
(a) Spectrogram of the original traditional microphone speech; (b) Spectrogram of the original radar sensor speech; (c) Spectrogram of the enhanced traditional microphone speech; (d) Spectrogram of the enhanced radar sensor speech.

Figure 3(a,b) shows the spectrograms of the original traditional microphone speech and radar sensor speech simultaneously collected from the same speaker, respectively. The content of the speech signal is the simple phrase “one-two-three-four”, which contains both voiced and unvoiced sounds. Figure 3(c,d) respectively shows the spectrograms of the enhanced recording. Original recordings contain some amount of noise, most of which were effectively removed by the spectral subtraction algorithm to yield enhanced recordings. The energies of both the traditional microphone speech and the novel radar sensor speech are distributed in a frequency range of 70–5,000 Hz. The spectrogram of the radar sensor speech is similar to that of the traditional microphone speech to the most minor details. In spectrograms (b) and (d), clear high frequency components indicate that the proposed sensor has good sensitivity to high frequency speech signals.

**Figure 4.**
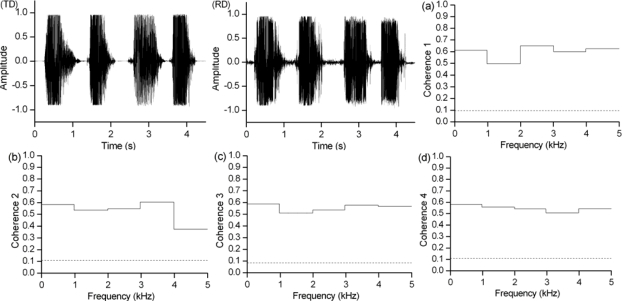
Plots **(TD)** and **(RD)** show the time domain signals of the traditional microphone speech and radar speech; Individual coherence between each of the corresponding words (“one”, “two”, “three”, and “four”) is shown in (a), (b), (c) and (d), respectively. The four horizontal dotted lines indicate the confidence level (α = 0.95).

Figure 4 presents TD and RD which are plots of the time domain signals of the enhanced traditional microphone speech and the enhanced radar sensor speech recordings, respectively. Moreover Figure 4 shows the average coherence over the 0–1, 1–2, 2–3, 3–4, and 4–5 kHz frequency bands between the traditional microphone speech signal and the radar sensor speech signal. Coherence between the same words of the same phrases simultaneously recorded by the novel radar sensor and the traditional microphone are plotted in Figure 4(a–d), with the horizontal dotted lines indicating the confidence level (α = 0.95). The TD and RD plots are very similar, and there is significant coherence between the two signals at most frequencies. The coherence of the same words indicates that the energy distribution of the radar sensor speech corresponds well to the energy distribution of the traditional microphone speech at most frequencies.

The results of the MOS tests are shown in Table 1. Columns G1 to G8 represent the listener groups, and the rows labeled Radar and Traditional show the mean opinion scores for the novel radar sensor speech and the traditional microphone speech, respectively. The mean opinion score of the novel sensor is higher than 4 in all groups, and the total perceptual mean opinion score is 4.4 ± 0.16, which indicates that the speech quality is between good and excellent.

**Figure 5.**
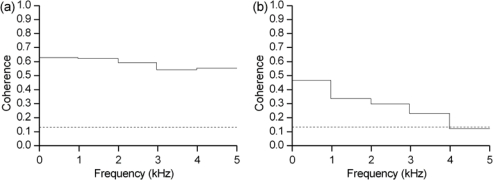
(a) Coherence between radar speech signals recorded with a wooden door barrier and those recorded without barriers. (b) Coherence between radar speech signals recorded through a brick wall barrier and those recorded without barriers.

Finally, the radar speech signals recorded with barriers were compared to those recorded without any barrier. Figure 5 shows the results of coherence analysis of these speech signals. The coherence between radar speech received without barriers and that with the wooden door is high, which indicates the sensor has good penetrability and can detect speech signals through wood barriers of a certain thickness. Between radar speech received without barriers and that through the sandwich brick wall, coherence is not as high, especially in the 4–5 kHz frequency band. These indicate that the novel speech radar sensor has penetrability for a brick wall barrier, although the speech quality is not perfect.

## 5. Discussion

At present, various speech detection techniques have been reported for different environments and for different applications. The capability of the MMW radar for detecting speech has been mentioned for pure experiments [9], clinic diagnoses, and speech processing applications [7,8,32]. However, complete radar sensor speech containing high frequency energy has not been determined, since studies about radar speech have paid more attention to low frequency speech signals for specific applications.

In this study, novel radar sensor speech and traditional condenser microphone speech were recorded simultaneously in a relatively quiet environment. The quality of the radar speech was comparable to that of traditional microphone speech and coherence between the two recordings shows that there is very little distortion of the speech detected by the proposed radar sensor, thus guaranteeing speech quality. Moreover, results show that the radar speech sensor can detect speech signals even when there is a thick barrier between the sensor and the sound source.

The energy of the novel radar sensor speech is distributed in both low and high frequency ranges. This could be attributed to the combined effects of a 35.5 GHz operating frequency and a superheterodyne receiver, which improves the detection sensitivity of the radar sensor for small vibrations caused by high frequency speech. Therefore, the novel radar sensor can detect high quality speech information. In theory, high quality information involves the interaction of EMW and AW information [4,33–35], and vibrational information of the skin and the speech organ [8]. In addition, the power of F0 of radar sensor speech is obtained mainly from vibrational information of the skin and the speech organ [36] for the good direction-sense of microwaves, which makes the sensor have high anti-jamming abilities in noisy environments [37].

The penetrability of the proposed novel radar speech sensor shows that the novel sensor may be preferable to other speech sensors for specific application. First, human subjects will feel more comfortable and relaxed because there is no need to attach the sensor to their body during operation. Electromagnetic radiation from the sensor also poses no safety threats, based on the standard for safety levels [38]. Second, during the penetration detection, the vibrations of barriers caused by sound pressure have a minor influence on radar speech for the barriers thickness and can be ignored. Thus, the sensor has potential for security applications, because it can be hidden from view behind non-metal materials of a certain thickness and still detects speech. Further studies should be performed to determine the potential significance of the sensor in other applications.

Through the experiments, some limitations of the proposed sensor were identified. Radar speech recorded in a quiet environment suffers more noise contamination, which could be attributed to the preprocessing circuit system. Improvements in the preprocessing circuit may reduce the recorded noise. Furthermore, the penetration capability of the proposed sensor varies for different barriers with different dielectric constants. The penetration depth in barrier for a certain wave frequency depends mainly on the dielectric constant and the loss factor [39], such that more studies on the penetration depth should be done to improve the performance of the novel sensor. Finally, the average coherence between the radar sensor speech and the traditional microphone speech in different frequency band is not uniform, which may be caused by the loss of some slightly harmonic components or some artifacts caused by the spectral subtraction algorithm. Therefore, a more appropriate antenna and an advanced algorithm may be able to give higher quality speech. More experiments are required to obtain optimum speech quality.

## Figures and Tables

**Table 1.** MOS of the radar sensor speech and traditional microphone speech.

**Microphone**	**G1**	**G2**	**G3**	**G4**	**G5**	**G6**	**G7**	**G8**
**Radar**	4.50	4.35	4.58	4.57	4.25	4.37	4.13	4.46
**Traditional**	5	5	5	5	5	5	5	5
